# Recent Advances in the Molecular Characterization of Circulating Tumor Cells

**DOI:** 10.3390/cancers6010595

**Published:** 2014-03-13

**Authors:** Lori E. Lowes, Alison L. Allan

**Affiliations:** 1 London Regional Cancer Program, London Health Sciences Centre, London, ON N6A 4L6, Canada; E-Mail: llowes@uwo.ca; 2 Department of Anatomy and Cell Biology, Schulich School of Medicine and Dentistry, Western University, London, ON N6A 5C1, Canada; 3 Department of Oncology, Schulich School of Medicine and Dentistry, Western University, London, ON N6A 4L6, Canada; 4 Lawson Health Research Institute, London, ON N6C 2R5, Canada

**Keywords:** cancer, metastasis, circulating tumor cells (CTCs), molecular characterization, prognosis, prediction

## Abstract

Although circulating tumor cells (CTCs) were first observed over a century ago, lack of sensitive methodology precluded detailed study of these cells until recently. However, technological advances have now facilitated the identification, enumeration, and characterization of CTCs using a variety of methods. The majority of evidence supporting the use of CTCs in clinical decision-making has been related to enumeration using the CellSearch^®^ system and correlation with prognosis. Growing evidence also suggests that CTC monitoring can provide an early indication of patient treatment response based on comparison of CTC levels before and after therapy. However, perhaps the greatest potential that CTCs hold for oncology lies at the level of molecular characterization. Clinical treatment decisions may be more effective if they are based on molecular characteristics of metastatic cells rather than on those of the primary tumor alone. Molecular characterization of CTCs (which can be repeatedly isolated in a minimally invasive fashion) provides the opportunity for a “real-time liquid biopsy” that allows assessment of genetic drift, investigation of molecular disease evolution, and identification of actionable genomic characteristics. This review focuses on recent advances in this area, including approaches involving immunophenotyping, fluorescence *in situ* hybridization (FISH), multiplex RT-PCR, microarray, and genomic sequencing.

## 1. Introduction

### 1.1. Metastatic Disease and Circulating Tumor Cells

Cancer is one of the leading causes of death in the United States, second only to heart disease. It is predicted that, in the United States alone, 1,660,290 individuals will be newly diagnosed and 580,350 individuals will die from this disease in 2013 [1]. The majority of cancer-related deaths occur as a result of metastasis. This lethality is largely attributable to our current lack of effective treatments for metastatic cancer [2,3]. One contributing factor to this is that metastatic lesions are highly heterogeneous when compared to their primary tumor counterparts [4,5,6,7,8,9,10]; however, the majority of treatment decision-making is currently based upon characteristics of the primary tumor. Although disease outcome is ultimately determined by metastatic spread, biopsy of metastatic lesions is often difficult to perform and can be a significant source of morbidity for patients. Therefore, it is currently not clinically feasible to subject patients to repetitive metastatic biopsies upon disease recurrence or progression, even if this approach could provide information that might improve treatment of metastatic disease. Unfortunately, this suggests that many patients are receiving sub-optimal treatment and therefore techniques that could better assess the characteristics of metastatic disease might enhance treatment efficacy and ultimately improve patient outcomes. 

Metastasis has been demonstrated to correlate with the presence of cancer cells in the peripheral blood circulation, hereafter referred to as circulating tumor cells (CTCs) [11,12,13]. The existence of CTCs has been known since the mid-1800s, when they were first reported by Thomas Ashworth, a resident physician at Melbourne Hospital. Upon autopsy of a patient with numerous (~30) subcutaneous tumors, Ashworth described these cells as appearing “exactly in shape, size, and appearance” to those seen in the primary lesions. Ashworth postulated that these tumor-like cells were cancer cells in the blood and that their existence could shed light on the “mode of origin” of numerous tumors in one individual [14]. Since the work of Ashworth in 1869, it has since been confirmed that the blood is a major route of transport for disseminating cancer cells, and it has been postulated that these CTCs might act as surrogate biomarkers of disease spread and patient outcome [11,12,13]. However, only recently has technological advancement allowed for detailed investigation of these cells and their consideration for use in the clinic.

### 1.2. Clinical Applications for CTCs

**Figure 1:**
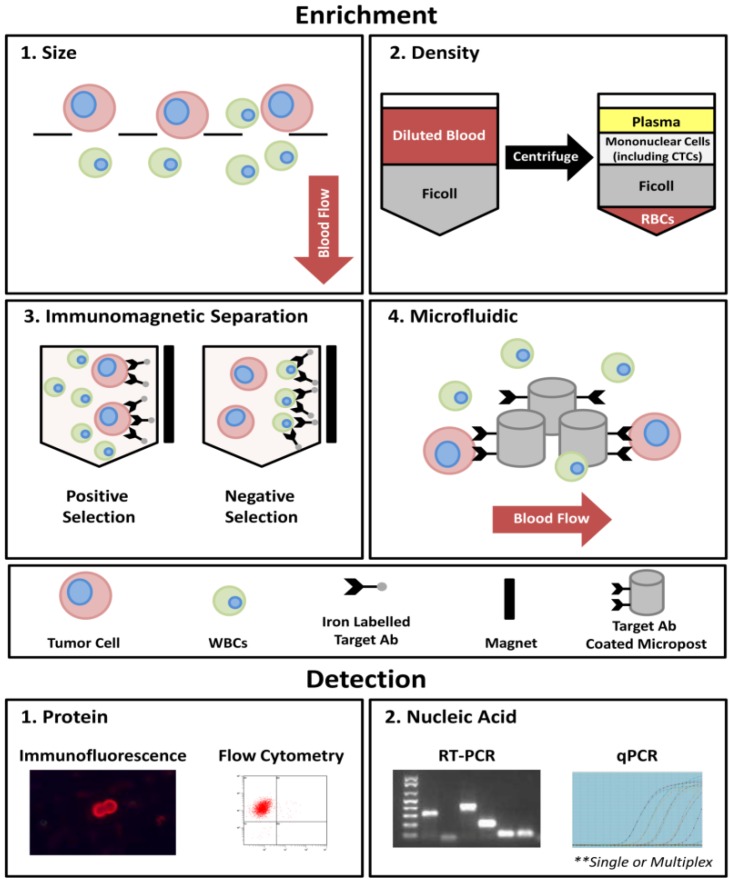
An overview of the most commonly utilized techniques for the process of CTC enrichment and detection. In general, four approaches currently exist for CTC enrichment: (**1**) size-based; (**2**) density-based; (**3**) immunomagnetic separation; and (**4**) microfluidic-based. Using size-based enrichment techniques, diluted whole blood is passed through a filtration device with specific sized pores (typically 8 µm). CTCs are captured based on differences in cell size between CTCs (typically >8 µm) and white blood cells (WBCs; typically <8 µm). Density-based enrichment utilizes Ficoll (or similar density gradient medium) to enrich for mononuclear cells (including CTCs) from other blood components. Immunomagnetic separation involves the use of iron-conjugated antibodies targeted toward CTCs (e.g., EpCAM; positive selection) or contaminating blood cells (e.g., CD45; negative selection) and incubation in a magnetic field. For microfluidic-based techniques, whole blood is slowly passed across a chip-based surface and isolated using either CTC targeted antibody-coated microposts (CTC Chip and iChip [21,22]), or dielectrophoresis (DEPArray [23,24]).Current CTC detection techniques use either a protein-based approach (*i.e.*, immunofluorescence or flow cytometry) expressed by whole cells or secreted proteins (EPISPOT assay [25,26,27]), or nucleic acid-based approaches such as RT-PCR or RT-qPCR, applied at the level of single genes or using a multiplex approach.

Thus far, the clinical uses of CTCs have focused mainly on enumeration. Due to the rare nature of CTCs, this process typically required both enrichment and detection steps (Figure 1). For enrichment, approaches include size or density-based techniques and/or immunomagnetic separation (*i.e.*, positive selection using epithelial-specific or tumor-associated markers; or negative selection using markers expressed by contaminating cells such as leukocytes). For detection, approaches include nucleic acid-based techniques such as reverse transcription polymerase chain reaction (RT-PCR), reverse transcription quantitative-PCR (RT-qPCR), microarray, or sequencing; and/or protein-based techniques such as immunofluorescence or flow cytometry using antibody-mediated detection. The advantages and disadvantages of each of these techniques have been extensively reviewed previously [15,16,17,18,19,20] and therefore will not be discussed here.

Despite the development of numerous CTC platforms using various combinations of the above enrichment and detection steps, capture of these cells is still technologically challenging due to their rare nature (~1 CTC per 10^5^–10^8^ white blood cells [28,29,30]), the potential presence of contaminating cells that can lead to false positive identification (*i.e.*, non-tumor epithelial cells, circulating endothelial cells), and the lack of a globally accepted marker for capture of all CTCs (*i.e.*, some CTCs may lose EpCAM (epithelial cell adhesion molecule)/CK (cytokeratin) expression as they enter the bloodstream via a process known as epithelial-to-mesenchymal transition [31]). In fact, currently, the only U.S. Food and Drug Administration (FDA) cleared system for CTC detection and enumeration in the clinic is the CellSearch^®^ system, (Janssen Diagnostics, LLC, Raritan, NJ, USA) developed in the early 2000s. This platform enriches for CTCs using positive immunomagnetic selection based on EpCAM, followed by immunofluorescent staining for CK 8/18/19; CD45; and the DNA dye DAPI (4',6-diamidino-2-phenylindole). Positive CTCs are identified, using semi-automated fluorescence microscopy, as cells with a CK^+^/DAPI^+^/CD45^−^ phenotype [32]. The CellSearch^®^ system is currently cleared for prognostic use in metastatic breast, prostate, and colorectal cancers, where the presence of ≥5 (breast [32] and prostate [33]) or ≥3 (colorectal [34]) CTCs in 7.5 mL of blood is correlated with poorer prognosis compared to patients with fewer CTCs in the same blood volume. Using this platform, CTC enumeration has been utilized not only to assess CTC number at baseline but also throughout the course of treatment and/or following completion of various treatment regimens. It has been demonstrated that CTCs are correlated with patient outcome and that the change in CTC number during treatment is predictive of therapy response, often sooner than currently utilized techniques such as imaging [33,35,36,37]. However, as described in the sections below, simple enumeration of CTCs fails to capitalize on their full potential as biomarkers of metastatic disease. 

### 1.3. CTCs as Surrogate Biomarkers for Metastatic Biopsy

As mentioned above, although the biopsy and subsequent molecular profiling of metastatic tissue would be ideal for determining appropriate interventional treatments for cancer patients upon disease recurrence or progression, this approach is typically not routinely feasible in the clinic. Therefore, molecular characterization of the cells that seed these metastatic lesions has been proposed as a surrogate for metastatic biopsy. For patients who have been heavily pre-treated with numerous lines of therapy, it is highly likely that the cancer cells that persist in the body are significantly different from those that originally existed in their primary tumor counterpart [4,5,6,7,8,9,10]. In addition, outside of the metastatic setting, CTCs may also demonstrate utility in circumstances where no primary tumor is available for characterization, or where the collected tissue is of poor quality and/or insufficient quantity. The molecular characterization of CTCs therefore holds great promise in terms of assessing disease status and will likely better represent the overall heterogeneity of disease at the time of necessary intervention.

Moving forward, molecular characterization of CTCs could provide an attractive and powerful alternative to metastatic biopsies; acting as a minimally invasive “real-time liquid biopsy” that can be repeatedly performed to allow assessment of genetic drift, investigation of molecular disease evolution, and identification of actionable genomic characteristics. This review focuses on recent advances in this area, including approaches involving immunophenotyping, fluorescence *in situ* hybridization (FISH), multiplex RT-PCR, microarray, and genomic sequencing. We will discuss the advantages and disadvantages of each approach and will summarize the most common aberrations that have been assessed using these techniques. In addition, we will use HER2 (human epidermal growth factor receptor 2), one of the most frequently assessed aberrations in CTCs, as an example of a “proof-of-principle” marker that demonstrates the potential clinical impact of CTC characterization. 

## 2. CTC Molecular Characterization Approaches

### 2.1. Protein-Based CTC Characterization Techniques

#### 2.1.1. Immunofluorescence

Immunofluorescence is the primary means by which CTCs have been interrogated at the protein level, using specifically targeted antibodies. A number of CTC enrichment techniques have been employed prior to immunofluorescent staining including immunomagnetic approaches (both positive and negative selection) [38,39,40,41,42,43,44,45,46,47,48,49,50], density gradient centrifugation [45], and microfluidic chip-based approaches [21]. Using immunofluorescence, CTCs have been characterized for expression of many markers including HER2 [38,39,41,42,43,44,45,46,47,48,49,50,51,52], EGFR (epidermal growth factor receptor) [38,53,54], androgen receptor [55,56], prostate specific antigen (PSA) [21], estrogen receptor [46,53], and progesterone receptor [53]. 

Thus far in the literature the gold standard CellSearch^®^ system is the most highly utilized system for CTC characterization at the protein level, using a single fluorescein isothiocyanate (FITC) fluorescence channel not required for CTC identification. Currently the CellSearch^®^ system has three commercially available markers that can be used on-system in combination with this platform to examine HER2, EGFR, or insulin-like growth factor 1 receptor (IGF-1R) expression on CTCs. In addition, the CellSearch^®^ system is amenable to the development of user-defined protein marker protocols for CTC characterization. However, it is noteworthy that the development of these protocols requires significant work-up and the use of rigorous controls to ensure proper optimization. In addition, the CellSearch^®^ system is a “closed” platform with little flexibility in terms of fluorophore selection and fluorescent channel availability. Currently CTC characterization using this system is limited to one additional marker. While this limitation in fluorophore availability is a hurdle that must be overcome by all protein-based platforms; several groups have developed systems that are more “open” in nature and therefore more amenable to extensive multi-marker CTC characterization (described below). 

A microfluidic herringbone chip-based assay known as the CTC Chip platform [21], the next generation CTC Chip platform, the iChip which combines microfluidic and magnetic cell sorting technologies [22], and a portable microfiltration platform [57] developed recently are excellent examples of “research-friendly” immunofluorescent techniques that allow flexibility in CTC characterization. These two platforms utilize different CTC enrichment methodologies to capture these rare cells, with the CTC Chip system relying on positive selection using anti-EpCAM coated microposts as blood is passed over the chip surface, *versus* the microfiltration system which utilizes size-based capture of CTCs. Thus far in the literature these two platforms have been exploited for CTC characterization, examining a variety of markers including PSA, M-30, thyroid transcription factor 1 (TTF1), Ki-67, and HER2 [18,21,57,58]. 

The advantages of utilizing immunofluorescence for CTC characterization include: (1) the ability to examine the presence or absence of expression, as well as protein localization and co-localization with additional proteins; (2) the ability to examine many proteins of interest simultaneously, limited only by the filter capacity of the investigators’ microscope; (3) the ability to visually confirm that expression is in CTCs and not contaminating cells; and (4) the ability to visualize variations in protein expression levels (it is important to note that this may also be seen as a disadvantage if not properly standardized). Several disadvantages also exist with regards to immunofluorescence techniques including: (1) limitations in assay sensitivity (*i.e.*, enough antigens need to be present to display a visible signal); (2) bleed-through from additional fluorescent channels can make interpretation of results confusing; and (3) using this approach, result interpretation can be more difficult to standardize (*i.e.*, what constitutes a true positive or negative signal), although automated CTC analysis approaches are evolving to help address this issue [45,51]. 

In the clinic, the primary benefit of immunofluorescence-based CTC characterization is the ability to identify the presence or absence of particular therapeutic target molecules, thereby expanding the availability of targeted therapies to patients who would previously be considered ineligible based upon the characteristics on their primary tumor. The limited availability of HER2 targeted therapies to breast cancer patients with HER2^−^ primary tumors is an excellent example of a setting in which CTC characterization could augment patient care. In particular, the detection of HER2^+^ CTCs in a patient with a HER2^−^ primary tumor could predict response to HER2 targeting agents and increase the availability of these personalized treatment options to patients. In the future, we envision serial CTC assessment at the protein level as a tool for predicting therapy response to specific targeting agents and facilitating evaluation of emerging drug resistance based upon the loss/downregulation of target molecules. 

#### 2.1.2. Flow Cytometry

Although immunofluorescence is a powerful tool for CTC characterization at the protein level, its primary limitation is that the data obtained using this approach is largely qualitative. Due to the highly heterogeneous nature of CTCs, quantitative analysis of these rare cells may be advantageous. Quantifiable flow cytometry assays are therefore an attractive alternative for protein-based characterization. In the clinic, flow cytometry has been proven to be an extremely powerful technology, with clinical flow cytometry being utilized in a number of disciplines, including hematology and oncology [59,60]. In general, flow cytometry has primarily been utilized for CTC enumeration; however, this technique is also an attractive method for multi-marker, on-system, molecular characterization of CTCs. Thus far in the literature, this technology has been utilized to examine the expression of EGFR and its phosphorylated counterpart, ALDH1 (aldehyde dehydrogenase 1), CD44, CD47, MET, and heparanase (HPSE) [61,62,63,64]. Simultaneous to on-system characterization, flow cytometry offers the ability to easily sort and collect characterized CTCs using fluorescence activated cell sorting (FACS) technology [62,63]. Additional advantages offered by flow cytometric methods include: (1) the ability to examine not only the presence or absence of marker expression but also to examine the *level* of expression in a measurable and quantifiable fashion; (2) the ability to easily perform multi-marker analysis on a single sample, limited only by laser and fluorescent filter set availability; and (3) ease of sorted sample collection and downstream characterization using other approaches. However, disadvantages also exist including: (1) limitations with regards to assay sensitivity even when combined with pre-enrichment steps [65,66]; and (2) the inability to visually confirm that results are from CTCs and not due to leukocyte contamination.

Moving forward, the use of flow cytometry for CTC characterization in the clinic could provide similar benefits as those recognized for immunofluorescent techniques. In brief, these techniques could provide valuable information regarding the expression of protein markers for targeted therapies and the detection of drug resistant phenotypes, as well as the added potential for performing multi-marker protein analysis with a quantifiable readout. When utilized clinically, this approach would be better equipped (relative to immunofluorescence) for assessing overall CTC heterogeneity and for identifying distinct CTC subpopulations. An example of this has recently been elegantly demonstrated by Baccelli *et al.*, who identified a CD44^+^CD47^+^MET^+/−^ CTC subpopulation that is enriched for metastasis-initiating cells [62]. In addition, flow cytometry would also allow for these subpopulations to be quantified, potentially providing information regarding patient prognosis [62]. However it is important to highlight that current limitations with regards to assay sensitivity restrict the use of this technique as a clinical assay at present, and advances in technology are needed to address this.

### 2.2. Nucleic Acid-Based CTC Characterization Approaches

#### 2.2.1. Fluorescence *in Situ* Hybridization (FISH)

At the genomic level, fluorescence *in situ* hybridization (FISH) has been utilized to interrogate CTCs for changes in individual genes, including gene copy number, gene rearrangement, and/or gene deletion; as well as chromosomal changes, such as select arm deletion or amplification [38,39,40,51,52,67,68,69,70,71,72,73]. Prior to FISH analysis, CTCs are typically enriched from whole blood, with the exception of one group that have demonstrated FISH analysis of CTCs without prior enrichment [74]. In the literature several enrichment techniques have been employed, including the CellSearch^®^ system [38,39,40,52,70,71,73], isolation by size of epithelial tumor cells (ISET) [67,69], density gradient centrifugation [38,68,73,75], OncoQuick [72], and microfluidic chip-based assays [38,68,75]. Following enrichment, isolated CTCs from metastatic breast, prostate, and lung cancers have been examined by FISH (either on-platform or after being cytospun onto charged glass slides) for several common genomic aberrations and amplifications including HER2 [38,39,51,52,68,70,75], anaplastic lymphoma kinase (ALK) [67,69], phosphatase and tensin homolog (PTEN) [73], androgen receptor [40,71,73], EGFR [38,40], and TMPRSS2:ERG (transmembrane protease serine 2:ETS-related gene) fusions [72]. FISH has previously been demonstrated to be a powerful tool in assessing genomic aberrations in the clinic in primary and metastatic lesions [76]. Therefore it is not surprising that this technique has several advantages with regards to CTC characterization, including: (1) the ability to assess the genomic characteristics of individual CTCs with visual confirmation; (2) the ability to assign easily defined cut-off/threshold values based on quantifiable ratios of mutation to parent chromosome; and (3) the availability of automated FISH enumeration systems. As with all techniques, FISH does present several limitations as well, including: (1) the underlying fact that FISH interrogates CTCs at the genomic level and therefore results may not truly reflect CTC phenotype at the functional protein level; and (2) FISH assessment does not provide information regarding markers whose regulation and/or function rely on epigenetic changes, phosphorylation, or appropriate protein localization.

Since the results of FISH analysis are not necessarily representative of cellular phenotype and/or target molecule expression at the protein level, it is likely that FISH technologies will demonstrate their greatest clinical benefit at the level of disease prognosis. An example of this is illustrated by Attard *et al.*, in their characterization of CTCs for hetero- or homozygous deletion of PTEN [73]. PTEN is involved in the phosphoinositide 3-kinase (PI3K) pathway, and inadequate inhibition of this pathway is associated with high Gleason score and tumor progression [77,78]. Retrospective analysis has demonstrated that PTEN deletion in primary tumors could stratify patients into different prognostic groups, with hetero- or homozygous PTEN deletion resulting in shorter time to biochemical relapse following surgery and earlier recurrence of disease when compared to those patients without deletion [79]. Although not investigated by Attard *et al.*, presumably PTEN status on CTCs could be utilized in the future for assessing disease progression throughout the course of disease. By assessing PTEN deletion status in CTCs at baseline or changes in PTEN status with repeated sampling, patients deemed at high risk of progression could be recommended for more aggressive treatment options earlier, thereby sparing patients the morbidity associated with ineffective therapies.

#### 2.2.2. Reverse Transcription Polymerase Chain Reaction (RT-PCR) and Reverse Transcription Quantitative PCR (RT-qPCR)

With regards to CTC analysis, RT-PCR has been utilized as a means to both detect the presence or absence of CTCs as well as a means for specific molecular characterization. The target transcripts or combinations of transcripts utilized for CTC detection are predominantly of either epithelial- or tissue-specific origin (*i.e.*, EpCAM, prostate specific membrane antigen [PSMA], mucin-1 [MUC-1]), and therefore presumably not transcribed by contaminating leukocytes. However, several groups in the literature have also published the use of RT-PCR for additional molecular characterization of these rare cells following CTC enrichment using the immunomagnetic AdnaTest; including assessment of HER2 [80], estrogen receptor [80,81], and/or progesterone receptor [80,81]. One major disadvantage that limits the use of RT-PCR in the characterization of CTCs is that, although assay sensitivity is high, specificity can be reduced as a result of illegitimate transcription and false positives. It is because of this limitation that many have chosen to utilize RT-qPCR in place of traditional RT-PCR for CTC characterization. The major advantage that RT-qPCR has over RT-PCR is the ability to set defined cut-offs, in the form of C_q_ values, to reduce false positives based on levels of illegitimate transcription observed in healthy donor blood samples. Utilizing this approach results in an assay that is not only highly sensitive but also highly specific. As with RT-PCR, this technique requires prior enrichment for CTCs, with the majority of studies utilizing the CellSearch^®^ system’s Profile Kit [82,83] or a similar immunomagnetic approach [84,85] for this task, examining a multitude of prognostic markers including (but not limited to) HER2, TWIST1, CD133, EGFR, MET and VEGFR2 (vascular endothelial growth factor receptor 2) [72,80,81,82,83,84,85,86]. The ability to multiplex this approach and examine multiple genes at once from a very small initial sample volume is a significant advantage that this technique offers. In addition, very recent studies have demonstrated that novel PCR approaches may also be useful in examining microRNA expression, methylation status, and single nucleotide mutations on CTCs [83,86,87]. However, RT-PCR and RT-qPCR also have several well recognized disadvantages, including: (1) the inability to visually confirm that signals obtained are from CTCs and not due to leukocyte contamination; and (2) analysis of single CTCs is still technologically challenging using this approach with few studies having published results from patient samples [84]. Therefore the majority of studies in the literature rely on pooled samples, which limits the ability to examine heterogeneity in marker expression across multiple CTCs in a single sample. 

Although both RT-PCR and RT-qPCR have been routinely employed for CTC detection and characterization [72,80,81,82,83,84,85,86], their widespread utility, especially with regards to detailed molecular characterization of heterogeneous CTC populations, is currently restricted by their limited capacity for single-cell analysis. Based on this current limitation, and the availability of a number of other excellent single-cell analysis techniques, at present we do not foresee this technique to be the primary CTC characterization choice for predicting targeted therapy response, drug resistance development, or prognosis in the metastatic setting. However one area in which we anticipate that these approaches will be very advantageous are clinical settings in which staging has demonstrated no macro-metastatic disease, also known as primary disease. In this clinical setting, blood analysis can yield very low numbers of CTCs [88], with the only way to combat this issue being the collection of larger blood volumes. Therefore characterization of CTCs in patients with primary disease is extremely challenging and extra care must be taken in obtaining the greatest amount of information from this small sample size. PCR approaches are beneficial in this regard as they allow for the amplification of these small samples and for multi-marker analysis allowing for the assessment of many potential targets at once. For example, using this approach, a pooled sample with isolated CTCs from a primary breast cancer patient could be assessed for expression of HER2, EGFR, estrogen receptor, progesterone receptor, and cancer stem cells markers simultaneously, thereby increasing the likelihood of obtaining useful CTC characterization information that could help direct patient care. Therefore, in the future we anticipate that RT-PCR and RT-qPCR approaches will demonstrate their greatest clinical benefit in the setting of early stage/primary disease, with the potential for widespread utilization in the metastatic setting based upon the optimization of single-cell analysis protocols.

#### 2.2.3. Microarrays

Both gene expression arrays and comparative genomic hybridization arrays (aCGH) have been used to characterize CTCs. Gene expression arrays provide information about samples at the RNA level, in particular the up/down regulation of suspected and novel transcripts; while aCGH provides information about samples at the DNA level, including copy number variations, specific mutational variants, or global genomic changes. Both techniques require that experimental samples be compared to appropriate control samples. Depending upon the information that one wishes to obtain, these controls will vary. For example, to obtain information regarding differences between CTCs (experimental) and primary/metastatic lesions (control), samples of each must be obtained and analyzed for differences using pre-determined cut-off values (*i.e.*, 1.5 fold change). Immunomagnetic enrichment [89,90,91] and density gradient centrifugation [92] have been the primary means utilized as upstream CTC enrichment techniques prior to microarray analysis. In the literature, microarrays have been primarily utilized to look for genetic signatures of aggressive disease and/or the identification of prognostic/diagnostic biomarkers of disease [90,92]. In addition, gene or copy number aberrations have been examined in CTCs [89,91,93]. The obvious advantages of array-based analysis include: (1) automated analysis; (2) the ability to set pre-determined cut-off values, thereby standardizing interpretation; (3) direct comparison of a multitude of disease settings (e.g., CTCs to primary/metastatic tumors, CTCs in treatment responders *versus* non-responders, CTCs at baseline *versus* following systemic treatment, *etc.*); and (4) the potential for novel biomarker identification and/or CTC gene signatures. In addition to the many advantages that this approach offers, several limitations also exist, including: (1) the necessity for specialized bioinformatics personnel for the analysis and interpretation of the massive amount of data that can be generated using this approach; (2) the necessity for validation of individually identified genes (gene expression arrays) using RT-qPCR; (3) cost; (4) difficulty in assessing sample purity to determine if results are from CTCs or contaminating leukocytes; and (5) limitations with regards to sensitivity that can make single cell analysis difficult, with few studies reporting on arrays using individual CTCs [93].

In the future, we anticipate that the most useful application of microarray-based approaches for CTC analysis may be in the area of prognosis and patient treatment stratification using CTC gene signatures. This approach has previously been demonstrated to be feasible when examining primary tumor tissue in breast cancer using the FDA approved MammaPrint^®^ Breast Cancer Test by Agendia (Irvine, CA, USA) [94,95]. Using this assay, primary tumor tissue is collected and subjected to array analysis to stratify patients into poor or good prognosis groups, and recommendations for aggressive (hormone therapy plus chemotherapy with or without trastuzumab) or less aggressive (hormone therapy alone) treatment, respectively, can be made based upon the results. Although this level of personalized care has not yet been met using microarrays on isolated CTCs, moving forward, microarray approaches may hold similar potential in this regard. 

#### 2.2.4. Sequencing

Until recently, the use of sequencing in clinical cancer genomics has presented significant logistical and economic challenges, due to the slow speed of sample processing and the high cost of sequencing. However the development of novel, next-generation sequencing technologies has renewed enthusiasm in the field of clinical cancer genomics [96,97,98,99]. Sequencing is an umbrella term that encompasses a number of different methodologies including traditional gene sequencing approaches (Sanger sequencing; pyrosequencing; MALDI-TOF sequencing; and targeted sequencing approaches such as allele-specific RT-PCR and qPCR melting curve analysis) and next-generation sequencing platforms (Roche 454™ pyrosequncing, Life Technologies SOLiD™ sequencing and Ion Torrent™ sequencing, the Illumina HiSeq™, the Helicos Heliscope™, Pacific Biosciences PacBioRS™, and Complete Genomics CGA™ platform), all of which have been reviewed previously [97,98,99,100,101,102,103,104]. Each technique has specific advantages and disadvantages, with all resulting in the acquisition of the base-by-base sequence information for a particular genome or target region within that genome. Sequencing technology is a powerful tool for the analysis of specific genomic aberrations, especially in the setting of cancer. It is important to note that this technique can be applied to both genomic DNA and transcribed RNA sequences in the form of cDNA. With regards to CTC analysis, sequencing tends to be applied more frequently at the level of RNA; however several studies have also interrogated CTCs at the DNA level [87,105,106,107]. In general, for processing at the RNA level, total RNA or mRNA is extracted from CTCs following enrichment using either immunomagnetic methods [108] including the CellSearch^®^ system [87,109], density gradient centrifugation [110], or microfluidic chip-based [111] approaches. Isolated RNA is then reverse transcribed into cDNA and PCR amplified using primers that are specific to the mutant/target region. Amplified mutations can be detected using either gel electrophoresis for known length transcripts, and/or analyzed with one of the several commercially available sequencing platforms mentioned above. For processing at the DNA level, instead, total DNA is extracted from CTCs, whole genome amplified using commercially available kits, and subsequently amplified via PCR using primers that are specific to the mutant/target region. As with RNA, the PCR product is then analyzed using either gel electrophoresis or a sequencing platform. Many studies in the literature have utilized these approaches to interrogate CTCs for a variety of single nucleotide changes in KRAS [87], BRAF [87], p53 [108], androgen receptor [111], TMPRSS2:ERG [111], PI3KCA (phosphatidylinositol-3-kinase, catalytic subunit α) [107], and EGFR [105,106]. 

One of the first reported studies examining the utility of CTC sequencing was reported by Maheswaran *et al.*, in their examination of EGFR activating and drug-resistant mutants in non-small-cell lung cancer patients [112]. Throughout the study this group not only demonstrated the presence of the primary EGFR activating mutation in CTCs but also the presence of a T790M mutation known to confer resistance to EGFR-targeted therapies. Using serial CTC analysis, it was additionally observed that the genotype of captured CTCs evolved throughout treatment and that the prevalence of the T790M resistance genotype increased throughout the course of therapy, suggesting that CTCs may be representative of the current state of disease. In a recent report by Heitzer *et al.*, single CTCs from metastatic colorectal cancer patients were assessed for a panel of 68 colorectal cancer-associated genes [107]. Using this approach, CTCs were shown to harbor mutations found in both the primary and metastatic lesions, metastatic lesions alone, and novel mutations not previously observed in either the primary or metastatic sites (termed private mutations). Subsequent ultra-deep sequencing of primary and metastatic sites often revealed the presence of these private mutations, previously missed by sequencing but captured by CTC analysis. In addition, many of the identified mutations were for actionable targets, with FDA-approved drugs currently available or being assessed for targeted treatment in ongoing clinical trials. 

The utilization of sequencing for CTC analysis has several advantages over other characterization techniques including: (1) the ability to identify single nucleotide alterations, since minor aberrations such as these can result in significant phenotypic changes and may be important for predicting response to select therapies; (2) results from sequencing are presented as either positive or negative and do not appear as gradations as with immunofluorescence; and (3) analysis can be automated to reduce interpreter bias. Sequencing techniques also have several marked disadvantages including: (1) limitations with regards to sensitivity that make single cell analysis difficult, with many groups reporting the need for a minimum of 50 or more CTCs for adequate results [87,108]; and (2) leukocyte contamination and the inability to visually confirm the source of amplified transcripts can lead to false positive/negative results. However, several groups have attempted to utilize single cell micromanipulation (selecting for CTCs based on immunofluorescent staining prior to the collection of DNA/RNA) [105] and/or adapted PCR protocols (e.g., nested PCR) [87] to combat these issues with promising results.

When considering clinical cancer genomics moving forward, care must be taken in discriminating driver mutations from so-called passenger mutations. As genomic instability is an underlying characteristic of cancer [113,114] one cannot assume that all mutations in a given sample are of equal importance. This is well exemplified by the great clinical benefit of trastuzumab for HER2-amplication in breast and gastric cancers but the lack of this benefit in ovarian and endometrial cancers [96,115,116,117]. In addition, the identification of actionable/druggable targets must be at the forefront of clinical cancer genomics. There is concern in this field that the genotyping of tumor tissue biopsies and/or CTCs may not be capturing *functionally* relevant information [118,119]. The reason for this concern centers on the fact that the cellular genotype is not necessarily reflective of the cellular phenotype and that sample contamination with normal tissue can lead to false negative results. We anticipate that the molecular characterization of CTCs will help to alleviate some of these concerns. Firstly, Heitzer *et al.*, have described a CTC sequencing approach for *single-cell analysis*, suggesting that contamination with normal cells may be reduced. Secondly, although the sequencing of CTCs does not change the fact that the readout is still at the level of the genome, we anticipate that, especially in cases in which metastatic lesions are inaccessible, that CTC sequencing will strengthen conclusions regarding mutations that are drivers *versus* those that are passengers as they may be present not only in the primary/metastatic lesion but also in the cells that were able to escape into the circulation. The conserved nature of these mutations may suggest an important functional contribution to disease progression. In addition, as demonstrated by Heitzer *et al.*, the sequencing of CTCs may identify relevant private mutations, present but not detected in tumor tissue [107]. 

In the future we anticipate that the greatest clinical benefit of the genomic sequencing of CTCs will be achieved when this approach is utilized to assess the genomic evolution of disease within a patient over time, and to quickly identify actionable target mutations that would make patients eligible for ongoing clinical trials, as demonstrated by Heitzer *et al.* [107]. As it is still unclear if genomic sequencing will provide *functionally* relevant information that can be applied for predicting targeted treatment response and overall patient outcomes, we foresee that this approach, at least in the near future, will likely not be utilized in isolation and instead used in combination with other phenotyping platforms such that firm conclusions can be drawn regarding clinical treatment decision making. 

### 2.3. General Considerations for CTC Characterization

Although there are currently a number of exciting methodologies available for CTC characterization and even more in development, careful consideration needs to be placed on which technique will produce optimal results for the molecular characteristic(s) under investigation, as it is likely that different aberrations will require different approaches. For example, the presence or absence of a particular marker may be sufficient for some targets, (e.g., estrogen/progesterone receptors) [120], however others may require the presence of particular single nucleotide substitutions (e.g., BCR-ABL mutations which confer drug resistance) [121], alternations to copy number (e.g., androgen receptor amplification) [122], aberrant localization (e.g., BRCA1 absent/reduced nuclear expression and association with aggressive phenotypes) [123], or specific functional activation (AKT phosphorylation) [124,125] in order to draw any conclusions regarding novel treatment options or patient outcomes. As not all approaches can provide this information, care must be taken in choosing the appropriate molecular characterization technique or combinations of techniques for each target. In addition, once chosen, the appropriate technique(s) needs to be validated and standardized before it can be considered for routine use in the clinic. This standardization needs to be implemented at both the level of procedure as well as at the level of interpretation. For example, data must be interpreted to determine if analysis of single cells is necessary or if a pooled result from all CTCs in an individual will suffice. If a single cell approach is chosen, clear-cut criteria must be set with regards to how many cells should be characterized with appropriate minimum or maximum values set. In addition, what constitutes a positive or negative signal must be appropriately defined; if the system is more graduated in nature (*i.e.*, low, medium, or high expression) these gradations need to be specifically defined, and if necessary automated systems need to be implemented to ensure that results are the same across laboratories. The considerations discussed in this section can all have a dramatic impact on the results obtained from individual studies and clinical trials. Therefore when comparing the current literature one must take into account the variety of approaches utilized and the effect these approaches may have on the reported results. These considerations and others have been extensively reviewed previously [17,126,127,128]. With these considerations in mind, in the following section we will utilize HER2 as a proof-of-principle marker to demonstrate the potential clinical significance of CTC molecular characterization. 

## 3. Clinical Significance of CTC Molecular Characterization: HER2 as a Proof-of-Principle Marker

**Figure 2:**
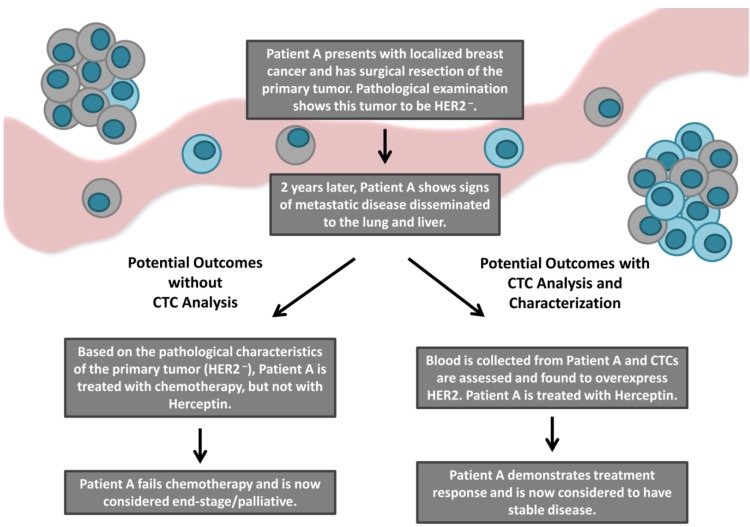
An overview of the current and potential patient outcomes following the incorporation of CTC molecular characterization into the clinic. The majority of cancer-related deaths result from the development of metastatic disease. Although metastatic lesions can be highly heterogeneous compared to their primary tumor counterparts, current treatment decision making is typically based on characteristics of the primary tumor, as routine metastatic biopsy is not clinically feasible. CTCs have been suggested as a surrogate to metastatic biopsy. Characterization of therapeutic target molecules such as HER2 on CTCs may increase the availability of targeted therapies (*i.e.*, the HER2 targeting agent Herceptin) to patients previously considered ineligible based upon the characteristics of their primary tumor. Ultimately, utilization of CTC analysis and characterization in the clinic may predict response to targeted therapies and improve patient outcomes.

Although a number of different molecular characteristics have been investigated on CTCs across numerous epithelial cancers, the aberration that has been most widely examined is HER2 in breast cancer. Therefore, in this section we will utilize HER2 as a proof-of-principle example to illustrate the potential clinical value of CTC characterization (Figure 2). 

### 3.1. The Role of HER2 in Breast Cancer and Clinical Assessment

HER2 is a proto-oncogene that is overexpressed, largely due to HER2 copy number amplification at the DNA level, in ~20%–25% of breast cancer patients [129]. HER2 overexpression is correlated with enhanced tumor aggressiveness, therapy resistance, and ultimately poor prognosis for patients [130]. Patients with primary tumors demonstrated to overexpress HER2 are eligible for treatment with HER2 targeting agents, trastuzumab (trade name Herceptin), lapatinib (trade name: Tykerb/Tyverb), and/or lertuzumab (trade name: Perjeta). Trastuzumab is a HER2 targeted interfering monoclonal antibody and has been demonstrated to provide therapeutic benefit in ~25% of HER2^+^ patients when utilized as a single agent and ~50% of HER2^+^ patients when used in combination with chemotherapy [131,132,133]. Lapatinib, a dual small molecule inhibitor of both HER2 and EGFR, and pertuzumab, a monoclonal antibody targeting a different HER2 epitope then trastuzumab, are both novel HER2 targeted therapies that have demonstrated promising results when used in combination with trastuzumab therapy [134,135,136].

The American Society of Clinical Oncology/College of American Pathologists (ASCO/CAP) currently recommends the following guidelines to assess HER2 status in primary tumor specimens [76]. Samples are considered HER2^+^ if any of the following are observed: an immunohistochemical (IHC) staining of 3+ (defined as uniform intense membrane staining of >30% of invasive tumor cells), a FISH ratio (defined as the number of HER2 gene signals to CEP17 gene signals) of >2.2, or a FISH result of >6 HER2 gene copies per nucleus. Samples are considered HER2^−^ if any of the following are observed, an IHC staining of 0 or 1+, a FISH ratio of <1.8, or a FISH result of <4 HER2 gene copies per nucleus. All other results are considered equivocal and require additional analysis to determine HER2 status as outlined in Wolff *et al.*, 2007 [76]. HER2 status is primarily determined at the time of diagnosis following primary tumor biopsy; however it is infrequently reassessed at the time of tumor recurrence in metastatic lesions. Until recently it was assumed that patients with HER2^−^ primary tumors would also have HER2^−^ metastatic lesions and therefore these patients were ineligible for treatment with HER2 targeted agents. However, recent evidence has demonstrated discordance in the HER2 status between primary and metastatic sites, suggesting that a subset of patients may be receiving sub-optimal treatment and could benefit from HER2 targeted therapies [7,137,138,139,140]. As biopsy of metastatic lesions can be difficult, many studies have instead examined HER2 status on CTCs as a surrogate assay.

### 3.2. Discordance in HER2 Expression between Tumor Tissue and CTCs

A number of studies have recently reported the characterization of CTCs for HER2 amplification and/or overexpression in breast cancer using a variety of techniques [38,39,41,42,43,44,45,46,47,48,49,51,52,53,68,70,75,80,89,141]. The vast majority of these studies have also compared HER2 expression to that of the primary tumor when available. Observed differences between primary tumors and CTCs in these studies have varied widely, with overall concordance ranging from 53% to 100%. Similarly, when considering only patients with HER2^−^ primary tumors and HER2^+^ CTCs or HER2^+^ primary tumors and HER2^−^ CTCs, discordance rates spanned an extremely wide range from 0% to 70% and 0% to 50% respectively [38,39,41,42,43,44,45,46,47,48,49,50,51,52,53,68,70,75,80,91]. These varied results are not surprising given that each study utilized different patient populations, different treatment regimens, different time points, and/or used different CTC enrichment, detection, and enumeration techniques. However, although all of these differences could add to these observed discrepancies, the greatest barrier to direct comparison of the published literature may be inconsistencies in HER2 evaluation and classification criteria. 

With regards to the methodology utilized for HER2 *evaluation* on CTCs, there is a general consensus that immunofluorescence and/or FISH are most appropriate, based on currently utilized primary tumor assessment techniques. However, with regards to HER2 *classification* in CTCs, a consensus does not currently exist. In general, there are two criteria that must be considered in determining HER2 positivity, including criteria for defining a HER2^+^ *CTC* (using either immunofluorescence and/or FISH); and criteria for defining a HER2^+^ *sample*. When using immunofluorescence to define HER2^+^ *CTCs*, 2 systems have been utilized; HER2^+^ CTCs defined as cells that express *any* HER2, or defined using a graded numbering system (0–3), based on HER2 staining intensity at the single cell level, similar to tumor tissue assessment [38,43,51,76]. Based on the potential subjectivity of this assay, automated intensity assessment would be optimal to reduce observer bias, as attempted by Ignatiadis *et al.*, (2011) [45]. When using FISH to define HER2^+^ *CTCs*, the standard protocol utilized for tissue samples is typically employed [76], with only minor variations in the HER2 to CEP17 ratio value chosen to define HER2 positivity, ranging from 1.8 to 2.2 [38,41,42,47,52,53,68,70,75]. When defining a HER2^+^ sample, several definitions have been utilized including the presence of *any* HER2^+^ CTCs; ≥50% of CTCs in a sample being HER2^+^ [39]; or the use of a weighted score based on the average HER2 value of each individual CTC (0-3) multiplied by the number of CTCs evaluated [38,46]. To add to this complexity, some studies have defined a minimum number of CTCs that must be analyzed in order for conclusions to be drawn [38,43]. Based on the variety of techniques utilized, it is clear that additional studies must be performed in order to develop a standard HER2 evaluation and classification criteria for CTCs. This definition can be subsequently applied in upcoming clinical trials in order to best interpret and compare results across studies.

### 3.3. Clinical Outcomes Based on HER2 Discrepancies

The effect of various systemic treatments (including HER2 targeted therapies) on the HER2 status of CTCs has been explored in several retrospective studies. Punnoose *et al.* recently examined HER2 expression in CTCs from metastatic breast cancer patients currently receiving treatment with Herceptin. In this small subset of patients (n = 6), CTC HER2 immunofluorescent H-scores (a metric defined by the authors, representative of HER2 expression) were not significantly different from the H-scores obtained for patients receiving non-HER2 targeting treatment regimens [38]. In contrast, Munzone *et al.* demonstrated in a study of advanced breast cancer patients with HER2 discordance between primary tumors and CTCs that most patients who demonstrated a switch in HER2 status were heavily pre-treated, many receiving 5–8 lines of therapy. In addition, Munzone *et al.* reported that all patients displaying HER2 discordance from HER2^+^ primary tumors to HER2^−^ CTCs were treated with Herceptin [44], suggesting the development of Herceptin resistance through acquired loss of HER2. With regards to therapy outcome, retrospective analysis of patients with HER2^−^ primary tumors and HER2^+^ CTCs has been described in several studies [44]. Meng *et al.* reported on four patients who had acquired HER2 amplification on CTCs with disease progression and who had been treated with Herceptin. Of these four patients, one showed a complete response, two showed a partial response, and one showed signs of progression [41]. The GeparQuattro trial aimed to detect and characterize CTCs before and following completion of neoadjuvant therapy in non-metastatic breast cancer patients. During this trial is was recognized that patients with HER2^+^ primary tumors and HER2^+^ CTCs treated with Herceptin had better patient outcomes then those with HER2^−^ CTCs. However, as this was not the primary endpoint of this trial this trend did not reach the level of statistical significance and therefore no conclusive results could be drawn from this subset of patients [50]. Hayashi *et al.* have reported in a retrospective analysis of metastatic breast cancer patients that the detection of HER2^+^ CTCs at first follow-up after treatment, but not at baseline, is predictive of poorer progression-free survival and overall survival. In addition, it was noted that when comparing patients with HER2^+^ CTCs that received Herceptin *versus* those that did not receive HER2-targeting therapies, 12.5% *versus* 66.7% of patients respectively succumbed to disease [47]. Finally, a randomized phase II study recently published by Georgoulias *et al.* demonstrated that patients with detectable CTCs in the adjuvant setting may benefit from secondary adjuvant treatment with trastuzumab when compared to observation alone [142]. Although this study did not stratify patients based on HER2 expression of CTCs, the high level of patients with HER2^+^ CTCs (~90% of eligible patients) suggests that this benefit may be as a result of the elimination of these chemo- and radiotherapy resistant cells.

Thus far, only one prospective clinical trial examining the utility of treatment stratification based on the HER2 status of CTCs has been completed [48]. This multicentre phase II trial aimed to evaluate the use of single-agent lapatinib (targeting both HER2 and EGFR) in metastatic breast cancer patients with HER2^−^ primary tumors and HER2^+^ CTCs. Study eligibility was assessed in 139 patients, with seven patients deemed to be eligible based on the requirement of a HER2^−^ primary tumor and ≥2 CTCs/7.5 mL with ≥50% of CTCs expressing HER2, as assessed using the CellSearch^®^ system. Of these seven patients, one experienced an adverse event during the treatment period and discontinued treatment. The other six patients demonstrated signs of progressive disease and therefore also discontinued treatment. Following these results the study was terminated. The authors suggest several explanations for the disappointing outcomes of this study, including heavy pre-treatment of these patient (≥4 lines of therapy) prior to lapatinib therapy; HER2 status on CTCs may not be indicative of the bulk of the metastatic lesions; and/or the definition used for eligibility may have been too strict thereby limiting the number of patients on the study and preventing efficacy analysis. Several upcoming trials are set to continue to investigate the HER2 status of CTCs and the use of CTCs as a liquid biopsy, including the TREAT CTC [143], DETECT III [144], CirCé01 [145], and the COMETI P2 [146] trials [147].

Although we concede that the results summarized here for HER2 are somewhat confounding, they do demonstrate that molecular characteristics of a patient’s disease can be discordant between primary lesions and CTCs, and that characterization of CTCs may be predictive of treatment response, at least when analyzed retrospectively. Therefore we suggest that further investigation into the utility of CTC molecular characterization in predicting response to targeted therapies is necessary. 

## 4. Conclusions: Current Limitations and Future Directions

The most prominent limitation that currently exists in the molecular characterization of CTCs is the low number of CTCs collected, especially from those patients with early-stage disease. These cells appear to be very delicate in nature and can be easily lost or destroyed during processing [70]. Considering that CTC populations tend to be quite heterogeneous, it is very difficult to draw conclusions about treatment if only a small population of cells are available for analysis. To combat this issue, research has begun to focus on the development of novel technologies for enhanced CTC capture as well as focusing on CTC characterization on-system, thereby reducing CTC loss when switching techniques. However, thus far the CellSearch^®^ system continues to be the only platform that is FDA-approved for use in the clinic. 

It is curious to note that in up to 35% of patients with various metastatic cancers, CTCs are undetectable despite the presence of widespread systemic disease [148]. This lack of detection has been proposed to be a result of the epithelial-to-mesenchymal transition, a well-documented process known to enhance cancer invasion, metastasis, and overall aggressiveness [149]. This transition has been associated with a significant reduction in epithelial markers (such as EpCAM) and a corresponding increase in mesenchymal markers [31]. Several studies have recently demonstrated that the presence of these mesenchymal markers in CTCs is correlated with poor prognosis, and that many of these cells lack expression of epithelial markers that would be necessary for their detection using many of the current CTC detection technologies [150,151,152,153,154,155,156]. This suggests that the standard definition of CTCs may be missing some of the most aggressive CTCs. Therefore, many emerging CTC detection approaches have sought to enhance capture of these more mesenchymal CTCs, suggesting that these cells may be of even greater importance for determining disease progression and ultimately patient outcome and therefore potentially the most relevant cells to characterize to determine subsequent treatment [57]. In addition to improving CTC capture, novel technologies are now beginning to focus on assays that demonstrate not only the presence of specified targets but also their functional activation (e.g., using phosphorylation status) in hopes of improving assay sensitivity for outcome prediction [157]. The current literature is also beginning to highlight the need for live CTC capture and subsequent functional analysis, with a specific focus on predicting treatment response by examining drug resistance on CTCs [93,111,158,159].

The current state of CTC molecular characterization is truly in its infancy. In this review we present a variety of methods that represent the current state of CTC molecular characterization, and describe the anticipated clinical benefits of each approach. In the future, the clinical application of CTC molecular characterization will benefit patients by aiding in the prediction of response to targeted therapeutics, the detection of drug resistant phenotypes, the identification of CTC subpopulations/gene signatures allowing for patient stratification, improved prognosis, and a thorough evaluation of patients for clinical trial eligibility. Unlike any approach utilized previously, CTC characterization will provide the added advantage of effectively monitoring patient disease evolution across time, providing a truly real-time liquid biopsy of disease. Ultimately, we anticipate that CTC molecular characterization will have a significant impact in the field of personalized medicine, and the development of novel CTC capture and characterization techniques will catapult the molecular characterization of CTCs to the forefront of personalized cancer care. 
